# A comprehensive study of the shielding ability from ionizing radiation of different mortars using iron filings and bismuth oxide

**DOI:** 10.1038/s41598-024-60188-2

**Published:** 2024-05-01

**Authors:** Wafa M. Al-Saleh, Mohamed Elsafi, Haifa M. Almutairi, Islam M. Nabil, M. A. El-Nahal

**Affiliations:** 1 College of Science and Health Professions, King Saud Bin Abdulaziz University for Health Sciences, P.O. Box 6664, 31982 Hofuf, Al-Ahsa Saudi Arabia; 2 King Abdullah International Medical Research Center, Hofuf, Al-Ahsa Saudi Arabia; 3 Physics Department, Faculty of Science, Alexandria University, Alexandria, 21511 Egypt; 4 Department of Physics, Faculty of Sciences, Umm Al-Qura University, 24382 Mecca, Saudi Arabia; 5 Physics Department, Faculty of Science, Fayoum University, Fayoum, Egypt; 6 Department of Environmental Studies, Institute of Graduate Studies and Research, Alexandria University, Alexandria, 21511 Egypt; 7 MEU Research Unit, Middle East University, Amman, Jordan

**Keywords:** Mortar, Attenuation coefficients, Gamma and neutrons, MNCP simulation, Experimental work, Physics, Nuclear physics

## Abstract

The current work discusses the radiation attenuation capability and different shielding characteristics of different mortar samples. The samples were prepared by replacing different percentages of fine aggregate with iron filling and replacing different percentages of hydrated lime with Bi_2_O_3_ (0–50 wt.%). The prepared mortar samples are coded as CHBFX where X = 0, 10, 30, and 50 wt.%. The mass and linear attenuation coefficient was determined experimentally using a narrow beam technique, where a high purity germanium detector, and different point gamma-ray sources (such as Am-241, Cs-137, and Co-60). The linear attenuation coefficient was also calculated using the Monte-Carlo simulation code and the online Phy-X/PSD software. The comparison of the three methods showed a good agreement in the results. The linear attenuation coefficient drops from 19.821 to 0.053 cm^−1^ for CHBF0, from 27.496 to 0.057 cm^−1^ for CHBF10, from 42.351 to 0.064 cm^−1^ for CHBF30, and from 55.068 to 0.071 cm^−1^ for CHBF50 at photon energy range from 0.015 to 15 MeV. The half-value layer thickness, tenth-value layer thickness, and mean free path of the prepared mortar composites were also calculated photon energy ranged from 0.015 to 15 MeV. The fast neutron removal cross-section of the prepared CHBFX mortar samples have values of 0.096 cm^−1^, 0.098 cm^−1^, 0.103 cm^−1^, and 0.107 cm^−1^ for the mortar samples CHBF0, CHBF10, CHBF30, and CHBF50, respectively. The results showed that the mortar sample with the highest iron filing concentration, CHBF50, provides the best protection against gamma rays and fast neutrons which could be used in the nuclear and medical fields.

## Introduction

Radiation technologies and applications have been spread widely in various industrial and medical fields, so the interest in protecting public and occupational workers has become critical. Radiation shielding technology is an important tool in radiation protection. Other measures like exposure time and distance between the source and individuals suffer many limitations when designing industrial or medical radiation applications. The concept of ALARA (as low as reasonably achievable) is a primary rule in radiation protection. All factors of radiation safety should be considered to fulfill it^1–3^. The radiation shielding technology creates low-cost, easily installed, and efficient radiation protection materials. Vast and various types of present and innovative materials are investigated by researchers, from traditional materials like lead, concrete, and glass to innovative composite materials reinforced by heavy metal oxide nanoparticles^4–6^.

Mortars and concrete are traditional shielding materials. They are characterized by their low cost and easy manufacture and installation. The advantage of being an environmentally friendly material also gives them a great advantage over toxic lead-based materials. The major drawbacks are the relatively low density and average atomic number of mortars and concrete constituents compared to lead-based or doped shielding materials.

The concrete and mortars are formed primarily by mixing different ratios of sand (fine aggregates) and cement or lime (binding material) inside an aquatic medium (water) with an additional component, in the case of concrete, which is the coarse aggregates. Mortars are lighter than concrete and are commonly used in masonry buildings to bridge the space between building units. Blocks of mortars can be utilized in radiation shielding, but due to mortar’s low density, as discussed before, there is a need to reinforce the mortar matrix with heavy elements or heavy element oxides^7,8^.

H Binici et. al. exhaustively examined the practicality of cement, Rilem sand, and eggshell industrial mortars^9^. Eggshell-containing mortars exhibited limited radioactive permeability because their linear absorption coefficient rose with the eggshell ratio. Egg shells can be used in radiation-effective areas. MI Sayyed et. al. examined mortar samples with varied Fe_2_O_3_ nanoparticle concentrations for radiation protection^10^. With increasing mortar thickness, photon transmission diminishes, according to I/I0 findings. Baltas et. al. studied the neutron and gamma-ray shielding capabilities of cement mortar were examined by adding minerals and ores with quantities from 0 to 30% as fine aggregate additions^8^. This study found that adding ores and minerals at 30% of the cement mass by weight did not significantly change the mortar samples’ gamma-ray and neutron attenuation properties. Pb–Zn and F–Ba were tested as mortar fine aggregate substitutes by Gallala et. al.^11^. Results showed that substitution materials affect mortar mechanical strength and gamma radiation shielding. Results reveal that these solid residues boost gamma radiation attenuation. F–Ba tailings mortars work better.

In the current study, iron filing waste will be reused and incorporated into the mortar matrix to partially replace the fine aggregates (sand). Since the disposal of iron filing waste is considered an environmental problem. Fine particles of iron filing wastes could transfer to the air, soil, and surface water, causing chemical and physical pollution. The reduction of leftover iron filing by utilizing it in the manufacturing of radiation shielding mortar will mitigate the adverse effects of iron filing waste disposal on humans and the environment. The expected improvement in radiation attenuation capabilities will be investigated at various concentrations of iron filing. This enhancement is due to an increase in the density and photoelectric absorption probability of mortar by increasing the concentration of iron filing through the material matrix^12,13^.

Furthermore, to increase the attenuation of designed mortars, heavy metal oxide of bismuth is added on account of lime in the mortar matrix. The known good attenuation properties, especially at absorption edges, have been studied and discussed by many literatures^14,15^. Mortar samples with various concentrations of iron filing and bismuth oxides will be studied to achieve the optimum mixture that produces the maximum attenuation without destroying the material matrix.

## Materials and methods

### Materials

#### Raw materials

**Figure 1:**
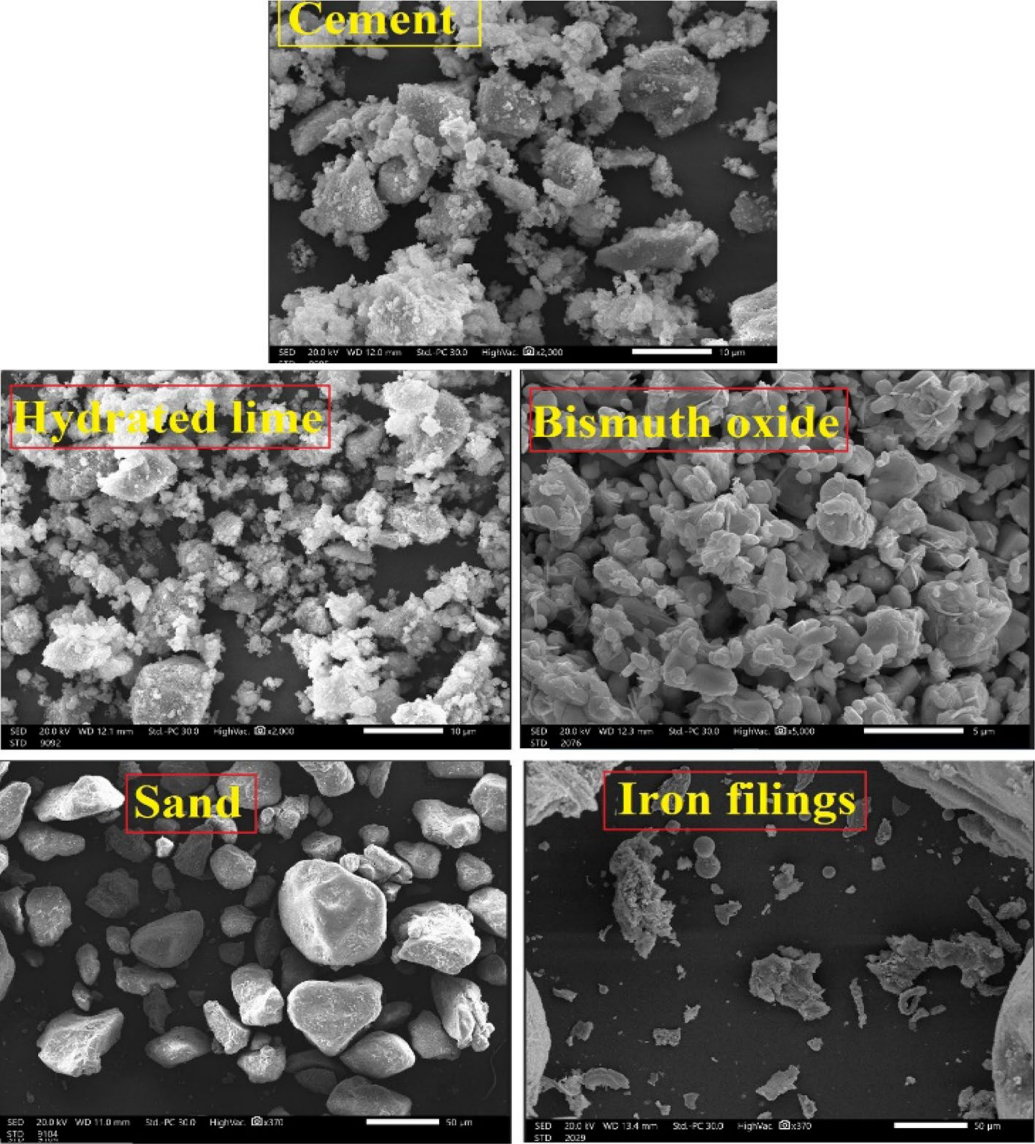
The SEM images of materials and oxides used in this work.

The materials used in this work are cement and hydrated lime as a binder and sand as a fine aggregate. The properties of these materials are reported in Table 1. The elemental composition, shape, and –particle size were performed using a scanning electron microscope (SEM-image), as listed in Table 2 and shown in Fig. 1.

**Table 1:** The properties of raw materials used in the present study.

Raw materials	Specific gravity	Average particle size (µm)
Cement	3.120	10
Hydrated lime	2.470	10
Sand	2.650	90
Iron filings	2.620	50
Bi_2_O_3_	8.900	2

**Table 2:** The elemental compositions of cement, iron filings, and sand.

Element	Mass, wt.%		
	Cement	Iron filings	Sand
C	4.050 ± 0.180	2.58 ± 0.130	2.66 ± 0.220
O	53.110 ± 0.530	1.660 ± 0.510	50.570 ± 0.490
Na	0.480 ± 0.050	–	0.670 ± 0.070
Mg	1.690 ± 0.050	0.500 ± 0.020	1.460 ± 0.080
Al	2.300 ± 0.060	–	3.900 ± 0.150
Si	10.070 ± 0.130	2.340 ± 0.310	31.920 ± 0.240
K	–	0.030 ± 0.040	1.260 ± 0.070
S	0.770 ± 0.040	0.010 ± 0.040	0.610 ± 0.050
Ca	25.110 ± 0.210	–	1.820 ± 0.090
Fe	2.410 ± 0.110	92.620 ± 0.150	4.110 ± 0.160

#### Mortar preparation

**Figure 2:**
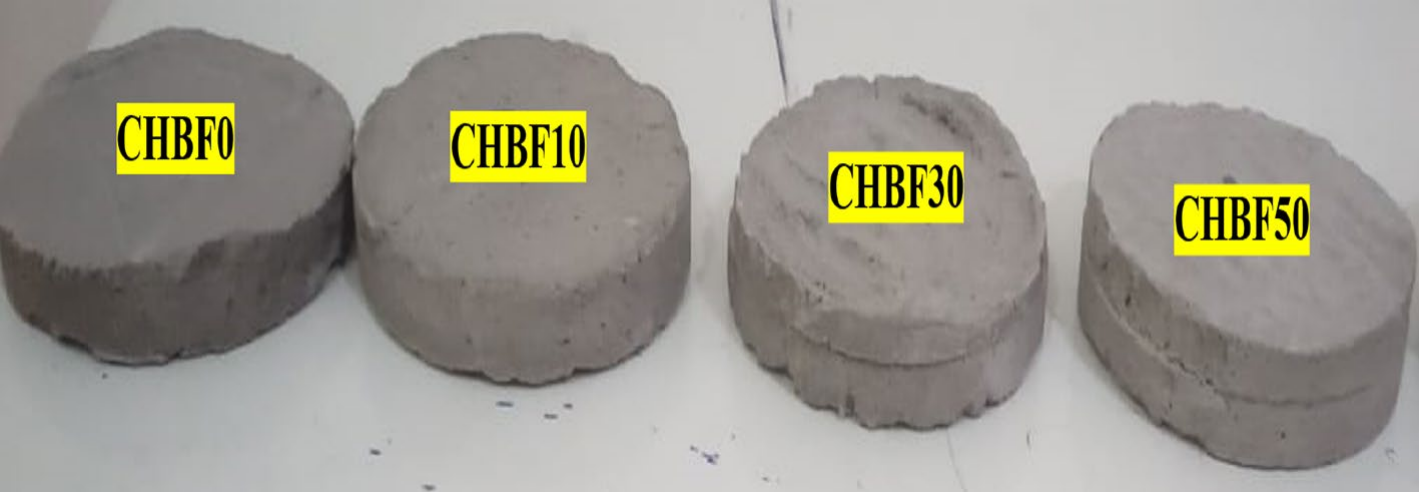
The mortar composites images.

The mortar composites were preictally prepared according to Table 3, Where iron filings were a partial substitute for sand, and in the same proportion, the bismuth oxide was a substitute for hydrated lime in proportions 0, 10, 30, and 50%. The materials were mixed manually in the proportions shown until they became completely homogeneous, after which water was gradually poured with mixing to form a slurry, which was then placed in cylindrical molds and left to dry^16–18^. Figure 2 represents the mortar composite images. Table 4 lists the elemental composition of the prepared mortar composites and their densities.

**Table 3:** Compositions of mortar composites in this work.

Replacement ratio (%)	Code	Cement (g)	Hydrated lime (g)	Bismuth oxide (g)	Sand (g)	Iron filings (g)	(w/c) ratio
0	CHBF0	200.0	50.0	–	1020.0	–	0.50
10	CHBF10	200.0	45.0	5.0	918.0	102.0	0.50
30	CHBF30	200.0	35.0	15.0	714.0	306.0	0.50
50	CHBF50	200.0	25.0	25.0	510.5	510.5	0.51

**Table 4:** the elemental composition of the mortar composites used in the radiation shielding investigation.

Sample ID	Elemental composition (wt.%)								Density (g.cm^−3^)
	Ca	O	Si	Al	Mg	Bi	H	Fe	
CHBF0	0.089	0.518	0.350	0.010	0.006	0.001	0.008	0.011	2.444
CHBF10	0.082	0.503	0.326	0.010	0.006	0.009	0.008	0.057	2.566
CHBF30	0.068	0.477	0.287	0.010	0.006	0.014	0.007	0.132	2.793
CHBF50	0.057	0.457	0.256	0.010	0.006	0.017	0.006	0.191	3.001

### Theoretical aspects

#### Gamma rays’ attenuation

From the count rate and sample thickness calculations, the µ (cm^−1^) can be estimated by the next law^10,19,20^:

1 $$\mu \left( {{\text{cm}}^{{ - {1}}} } \right) = \frac{1}{t} \ln \frac{{I_{0} }}{I }$$

The other essential attenuator factors, such as Half-value layer thickness (HVL), tenth-value layer thickness (TVL), and mean free path (MFP), are discussed in^19,20^ and can be expressed by the following law^21,22^:

2 $$HVL = \frac{Ln \left( 2 \right)}{\mu }$$

3 $$TVL = \frac{{Ln \left( {10} \right)}}{\mu }$$

4 $$MFP = \frac{1}{\mu }$$

The effective atomic number (Z_eff_): can be computed as :

5 $${\text{Z}}_{{{\text{eff}}}} = \frac{{\mathop \sum \nolimits_{i } f_{i} A_{i} \left( {\mu_{m} } \right){\text{i}}}}{{\mathop \sum \nolimits_{i } \frac{{A_{i} }}{{Z_{i} }}\left( {\mu_{m} } \right){\text{i}}}}$$

where* f*_*i*_ denotes the target element’s fractional abundance. The average atomic mass of any material is ∑*f*_*i*_*A*_*i*_ structure. Z_i_ denotes the atomic number.

#### Neutrons attenuation

A medium’s fast neutron removal cross-section (FNRCS, Σ_R_) is a typical way to describe its neutron-slowing properties. The linear attenuation coefficient defines the interaction of photons with matter; the removal of fast neutrons by materials can be seen as an analog of this (Σ_R_, cm^−1^). Also, the following formulas were used to find the half value layer (HVL_FNRCS_) and relaxation length (λ_FNRCS_) according to the neutrons calculations for the materials. The relaxation length is the average distance that a fast neutron can move before it interacts with the medium^23,24^:

6 $$HVL_{{{\text{FNRCS}}}} = \frac{ln2}{{{\Sigma R}}}$$

7 $$\lambda_{{{\text{FNRCS}}}} = \frac{1}{{{\Sigma R}}}$$

### Methods

#### Experimental attenuation measurements

**Figure 3:**
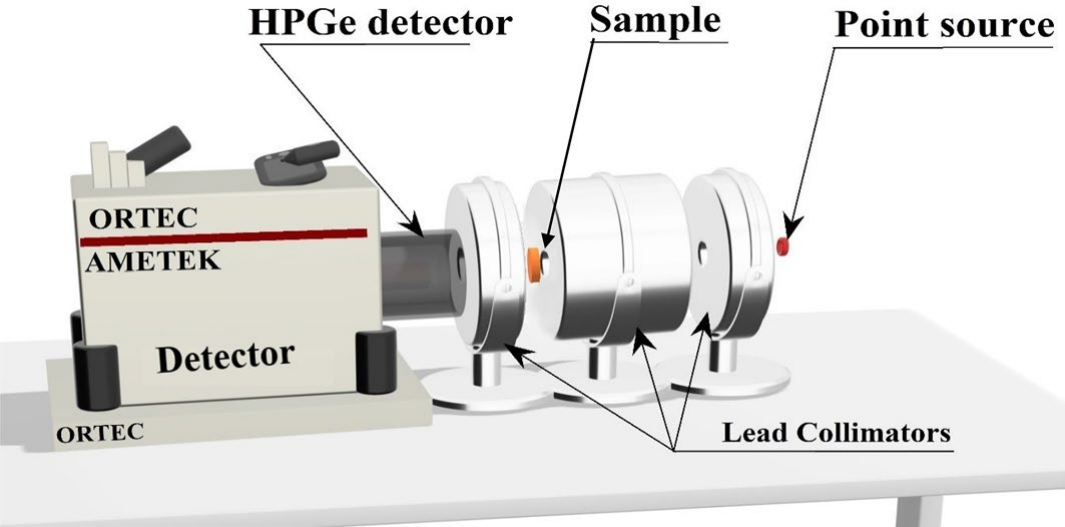
The experimental setup of the attenuation measurements.

The attenuation properties of the present mortar composites were tested experimentally using an ORTEC HPGe detector and different γ-point sources^25–27^. The experimental configuration of the narrow beam transmission system for the mortar composites is schematically shown in Fig. 3. The HPGe detector was calibrated using certified γ-point sources of the radionuclides: Am-241, Cs-137, and Co-60 of 0.9 µci which also used in the investigation of the gamma rays’ attenuation^28–30^. The γ-point sources were measured on the front face of the ORTEC HPGe detector for 3600 s. The experimental setup is optimized, with less than 1.5% dead time and <  ± 0.5% peak count rate uncertainty^31,32^. The count rate for each photon energy was measured using the gamma spectroscopy software Gamma Vision (5.3v)^31^ in case the absence (${I}_{0}$) and presence ($I$) of mortar composite of thickness ($t$) while maintaining all other conditions.

#### MCNP simulation

**Figure 4:**
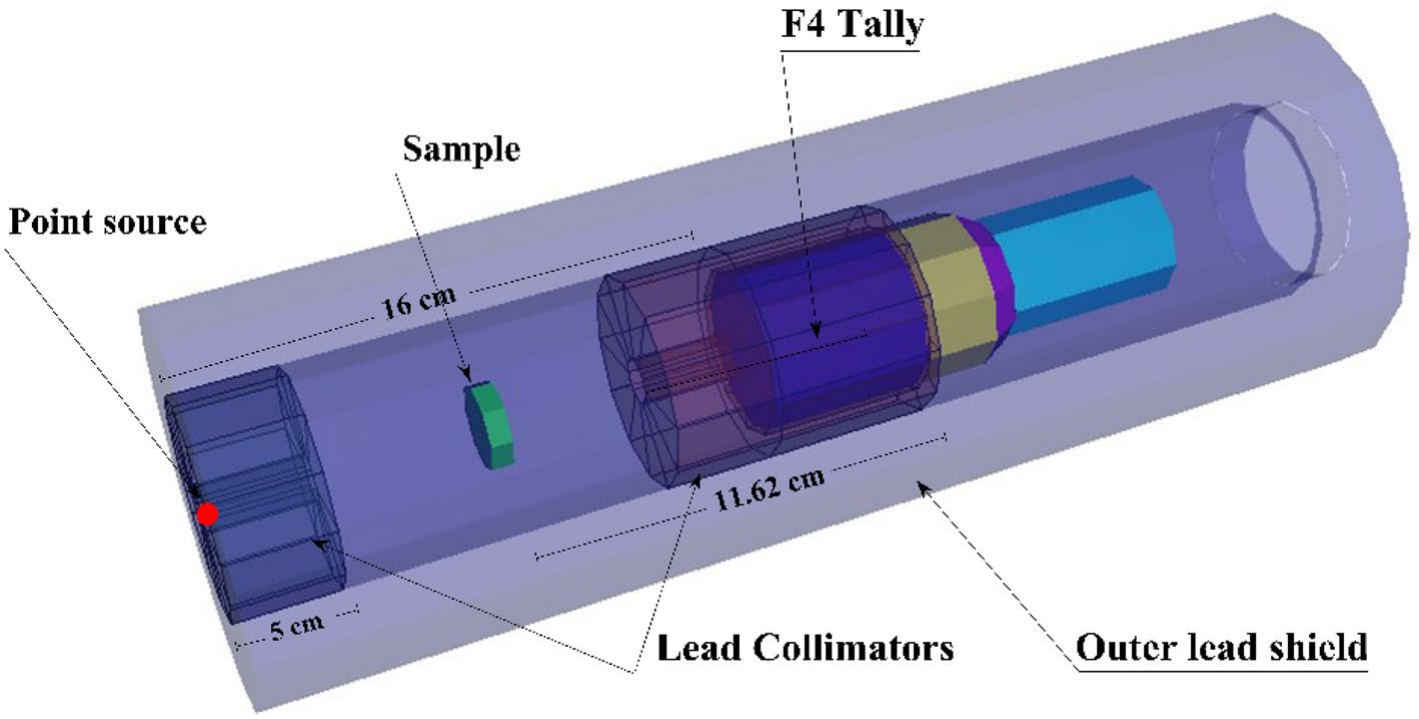
A 3D dynamic view of the used radiation attenuation simulation system for the CHBFX mortar samples.

Monte-Carlo for nature particles code MCNP5 (MC) was used to simulate the irradiation of the studied mortar samples CHBFX where x = 0, 10, 30, and 50 wt% in the energy range from 0.015 to 15 MeV. Taking into account the laws of physics interaction (photoelectric (PEE), Compton scattering (CSE), and pair formation processes (PPE), it stimulates the passage of neutrons and gamma photons^33–35^. Accurate information (source dimensions, source-to-detector distance, geometry, elemental chemical composition, etc.) must be provided in the input files used by MC, as seen in Fig. 4. In this experiment, every possible factor has been accounted for. Input files for MC were written in text format^36,37^. Six parts were detailed in the Text file: the radioactive source, the primary γ-rays collimator, the cubic sample, the secondary γ-rays collimator, and the detector. The radiation source was positioned inside the back of a lead collimator of the primary γ-rays and positioned 16 cm from the detector. For each input file, a point source of gamma rays with energy between 0.015 and 15 MeV was determined to be an SDEF mono-energetic beam^38^. A neutron source was described as a watt fission spectrum for fast removal cross-section attenuation. The samples were created as a cylinder layer positioned in the source to detector the distance. Additionally, the chemical composition and density of the investigated samples were entered into the material card. The detector was configured inside a lead collimator of the secondary γ-rays. Using the command F4:P/F4:N, one can calculate the typical path traveled by incident gamma rays and neutrons from created sources. The created cells were surrounded by an outer lead shield cell. The computations took roughly 12 min each run for a total of (90) input files. They were performed on a core i5-2.3 GHz processor with many NPS (10^7^) histories for each file to achieve random statistical errors of less than (1%)^34,35^.

#### Phy-X/PSD software.

Shielding and attenuation variables for the investigated material compositions, dosimetry, etc., can all be calculated with the help of the web-based tool Phy-X/PSD software (PhyX)^39^. Many calculations were performed using the PhyX input file, including those for the mass attenuation coefficients (*µ*_*m*_), etc. MeV’s elemental composition, densities, and energy ranges were added as PhyX input parameters^40^. The relative differences (Div., %) were also calculated by comparing the PhyX results for the CHBFX mortar samples with the MC results^41,42^:

8 $${\text{Div}}. \, \left( \% \right) \, = \left| {\frac{MC - PhyX}{{MC}}} \right| \times 100,$$

## Results and discussion

**Figure 5:**
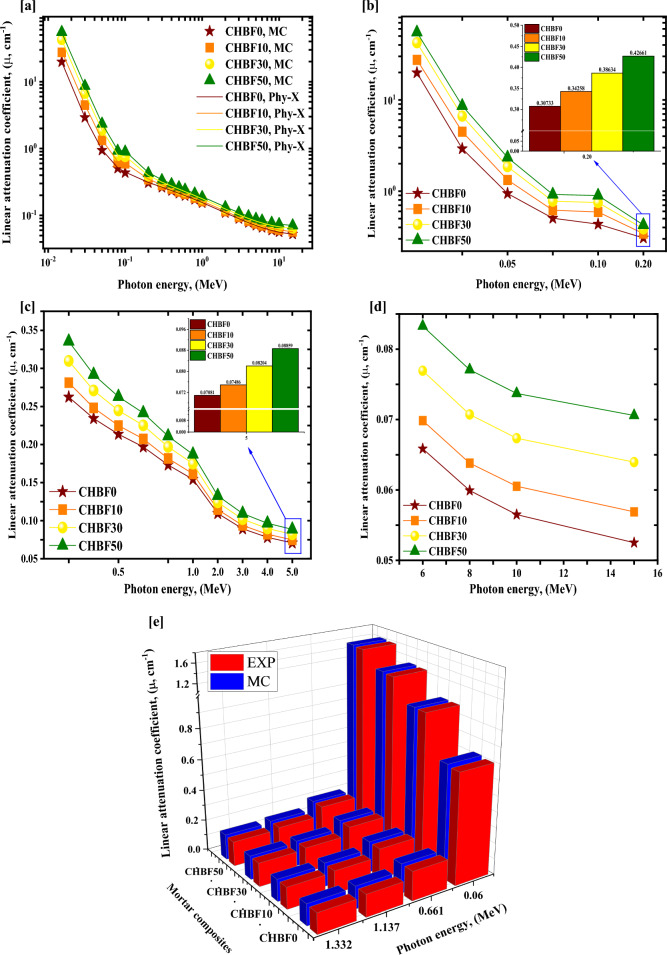
(**a–e**) Influence of gamma-ray energy on linear attenuation coefficient (**a**) obtained from MC and Phy-X, (**b**) due to photo-electric, (**c**) and (**d**) due to compton scattering regions, and (**e**) obtained from EXP and MC vs. photon energy for the CHBFX mortar samples.

Figure 5a–e represents the *µ* of the four synthetic CHBFX mortar samples. Figure 5a represent the *µ* of the four synthetic CHBFX mortar samples obtained by using MC code and PhyX software. The values of simulated µ are in good agreement with the values calculated by PhyX with a maximum relative difference of 3.826%, as listed in Table 5. Also, Fig. 5a illustrates that the µ decreases as the energy levels increase, a general trend observed across all materials. It is consistent with the behavior of radiation. This trend is particularly evident for the data where *µ* drops from 19.821 to 0.053 cm^−1^ for CHBF0, from 27.496 to 0.057 cm^−1^ for CHBF10, from 42.351 to 0.064 cm^−1^ for CHBF30, and from 55.068 to 0.071 cm^−1^ for CHBF50 at photon energy range from 0.015 to 15 MeV. As realized in Fig. 5b, There is a strong decrease in the *µ* values for the Synthetic mortar CHBFX samples due to PEE interaction, which has cross-section changes with ${E}_{\gamma }^{-4.5}$^43^. As a result, the photon-electron interactions and values decreased alongside the cross-section of interactions caused by the enrichment of photon values. The enhancement of the E_γ_ values between 0.015 and 0.200 MeV causes a strong exponential decreasing trend from 19.821 to 0.307 cm^−1^ for CHBF0, from 27.496 to 0.343 cm^−1^ for CHBF10, from 42.351 to 0.386 cm^−1^ for CHBF30, and from 55.068 to 0.427 cm^−1^ for CHBF50. The CHBF50 mor,tar sample has the highest values of the μ in this region due to the high concentration of iron filling (50 wt%) and its high density (3.001 g.cm^−3^). In addition, as shown in Fig. 5c, the values in the Eγ interval of 0.3–5 MeV decline exponentially as Eγ increases above 0.200 MeV. The CSE interaction with changes in cross-section caused by E_γ_^−1^ is blamed for the exponential decline^44,45^. It is explained by higher energy photons’ lower propensity to interact with the material’s atoms results from their greater velocity. As a result, when energy increases, the likelihood of photon absorption falls, and the likelihood of photon scattering increases^46^. The enhancement in Eγ values was linked to a smooth decrease in the cross-section with drops in the quantity of photon-electron interactions, followed by a smooth drop in the µ values^47,48^. The reduction in the µ values was from 0.262 to 0.071 cm^−1^ for CHBF0, from 0.281 to 0.075 cm^−1^ for CHBF10, from 0.310 to 0.082 cm^−1^ for CHBF30, and from 0.336 to 0.089 cm^−1^ for CHBF50 with raising the Eγ values between 0.3 MeV and 5 MeV. Also, still there is a little reduction due to the CSE interaction with cross-section changes with $E_{\gamma }$. The µ were from 0.066 to 0.053 cm^−1^ for CHBF0, from 0.070 to 0.057 cm^−1^ for CHBF10, from 0.077 to 0.064 cm^−1^ for CHBF30, and from 0.083 to 0.071 cm^−1^ for CHBF50 with raising the E_γ_ values between 6 and 15 MeV as showed in Fig. 5d.

**Table 5:** Linear attenuation coefficient (μ, cm^−1^) of the CHBFX mortar samples via MC and PhyX at different photon energies.

Photon energy, (MeV)	Linear attenuation coefficient (μ, cm^−1^)											
	CHBF0			CHBF10			CHBF30			CHBF50		
	PhyX	MC	Div.%	PhyX	MC	Div.%	PhyX	MC	Div.%	PhyX	MC	Div.%
0.015	19.686	19.821	0.678	54.560	55.068	0.923	42.055	42.351	0.698	54.560	55.068	0.923
0.03	2.925	2.919	0.216	8.672	8.620	0.605	6.673	6.639	0.512	8.672	8.620	0.605
0.05	0.957	0.942	1.562	2.376	2.342	1.476	1.885	1.857	1.535	2.376	2.342	1.476
0.08	0.518	0.506	2.420	0.949	0.921	3.038	0.800	0.776	2.994	0.949	0.921	3.038
0.1	0.444	0.435	2.084	0.917	0.896	2.376	0.768	0.750	2.397	0.917	0.896	2.376
0.2	0.311	0.307	1.346	0.438	0.427	2.611	0.396	0.386	2.373	0.438	0.427	2.611
0.3	0.264	0.262	0.798	0.342	0.336	1.875	0.315	0.310	− 1.609	0.342	0.336	1.875
0.4	0.235	0.234	0.356	0.296	0.292	1.282	0.274	0.271	1.142	0.296	0.292	1.282
0.5	0.214	0.214	0.206	0.266	0.263	1.138	0.247	0.245	1.008	0.266	0.263	1.138
0.6	0.198	0.197	0.364	0.244	0.241	1.011	0.227	0.225	0.913	0.244	0.241	1.011
0.8	0.173	0.173	0.104	0.213	0.211	0.717	0.198	0.197	0.671	0.213	0.211	0.717
1.0	0.156	0.154	0.936	0.190	0.187	1.775	0.178	0.175	1.629	0.190	0.187	1.775
2	0.109	0.109	0.129	0.134	0.133	0.802	0.125	0.124	0.668	0.134	0.133	0.802
3	0.089	0.089	0.320	0.110	0.109	0.457	0.102	0.102	0.389	0.110	0.109	0.457
4	0.078	0.078	0.461	0.097	0.097	0.304	0.090	0.090	0.181	0.097	0.097	0.304
5	0.071	0.071	0.432	0.089	0.089	0.237	0.082	0.082	0.130	0.089	0.089	0.237
6	0.066	0.066	0.365	0.083	0.083	0.231	0.077	0.077	0.076	0.083	0.083	0.231
8	0.060	0.060	0.593	0.077	0.077	0.100	0.071	0.071	0.091	0.077	0.077	0.100
10	0.056	0.056	0.625	0.074	0.074	0.166	0.067	0.067	0.139	0.074	0.074	0.166
15	0.052	0.053	0.542	0.071	0.071	0.111	0.064	0.064	0.124	0.071	0.071	0.111

Also, the µ values were calculated from the experimental measurements of the HPGe mentioned above detector system at γ-energy lines 0.060 MeV, 0.661 MeV, 1.173 MeV, and 1.332 MeV for the synthesis mortar CHBFX mortar samples. For the fabricated mortar samples, the simulated values obtained from the MC code were compared with those obtained from the experimental work as shown in Fig. 5e and listed in Table 6. With maximum relative differences of 3.112%, the comparison demonstrates agreement between the experimental and simulation calculations. The synthesis mortar thickness, densities, and chemical composition are srces of the variations. The maximum experimental errors in attenuation coefficients for γ-ray attenuation measurements were evaluated using the following error formula^49^:

9 $$\Delta \mu = \frac{1}{x}\sqrt {\left( {\frac{\Delta Io}{{Io}}} \right)^{2} + \left( {\frac{\Delta I}{I}} \right)^{2} + \left( {\frac{\Delta x}{x}} \right)^{2} + \left( {{\text{ln}}\left( {\frac{\Delta Io}{I}} \right)} \right)^{2} }$$

**Table 6:** An MC simulation comparison of µ values with those obtained from experimental measurements with the uncertainty for the CHBFX mortar samples.

Photon energy, (MeV)	CHBF0			CHBF10			CHBF30			CHBF50		
	EXP	MC	Div. (%)	EXP	MC	Div. (%)	EXP	MC	Div. (%)	EXP	MC	Div. (%)
0.060	0.709 ± 0.0011	0.722	2.520	0.969 ± 0.0021	0.972	0.250	1.287 ± 0.0013	1.309	1.990	1.599 ± 0.0010	1.621	1.480
0.661	0.187 ± 0.0015	0.189	1.180	0.196 ± 0.0011	0.199	1.430	0.213 ± 0.0017	0.216	1.640	0.231 ± 0.0015	0.231	0.830
1.137	0.142 ± 0.0009	0.144	0.910	0.146 ± 0.0009	0.151	3.160	0.161 ± 0.0008	0.163	2.120	0.171 ± 0.0012	0.174	2.920
1.332	0.131 ± 0.0013	0.135	3.110	0.139 ± 0.0018	0.141	1.410	0.152 ± 0.0012	0.153	0.840	0.159 ± 0.0016	0.164	3.110

; where; I is the transmitted γ-ray intensity, Io is the incident intensity of neutrons or γ-rays, while x is the mortar sample’s thickness.

From the obtained results,** t**he *µ* values for CHBF50 are generally the highest among the other mortar samples due to the increase of iron filling concentration doping (50 wt%), its high density (3.001 g.cm^−3^), and the high effective atomic number of the iron element (Z = 26).

**Figure 6:**
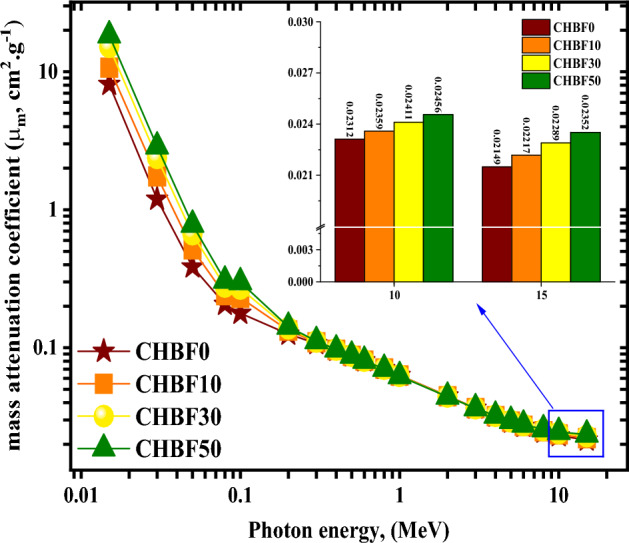
Influence of gamma-ray energy on mass attenuation coefficient vs. photon energy for the CHBFX mortar samples.

On the other hand, the *µ*_*m*_ for synthesizing CHBFX mortar samples takes the same behavior as the *µ.* The decrease in the µ_*m*_ values was from 8.110 to 0.021 cm^2^.g^−1^ for CHBF0, from 10.716 to 0.022 cm^2^.g^−1^ for CHBF10, from 15.162 to 0.023 cm^2^.g^−1^ for CHBF30, and from 18.348 to 0.024 cm^2^.g^−1^ for the CHBF50 sample with raising the E_γ_ values between 0.015:15 MeV as seen in Fig. 6.

**Figure 7:**
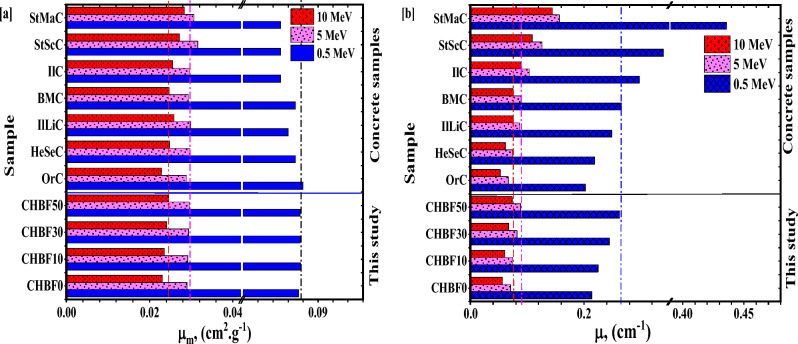
(**a,b**) The attenuation coefficients (**a**) *µ*_*m*_, (cm^2^.g^−1^), and (**b**) *µ*, (cm^−1^) for the CHBFX mortar samples compared with some reference concrete samples.

Figure 7a,b shows a comparison of the *µ*_*m*_ and *µ* between the CHBFX mortar samples and those of some commercial concrete samples (Ordinary concrete (OrC), Hematite-serpentine concrete (HeSeC), Ilmenite limonite concrete (IlLiC), Basalt-magnetite concrete (BaMaC), Ilmenite concrete (IlC), Steel-scrap concrete (StScC), and Steel magnetite (StMaC) at chosen energies 0.5, 5 and 10 MeV^50,51^. At 0.5 MeV, the *µ*_*m*_ of the CHBFX mortar samples have higher values than those of the compared concrete samples except the OrC sample. At 5 MeV, the *µ*_*m*_ of the CHBFX mortar samples have higher values than those of the compared concrete samples except for StScC and StMaC samples. At 10 MeV, the *µ*_*m*_ of the CHBF50 mortar sample has a higher value than those of the compared concrete samples. At 0.5, 5, and 10 MeV, the *µ* of the CHBF50 mortar sample has a higher value than those compared concrete samples for the samples IlLiC, HeSeC, and OrC.

Half-value layer thickness (HVL), tenth-value layer thickness (TVL), and mean free path (MFP) are common measures of radiation shielding effectiveness. They also reveal whether or not the shielding material is sufficiently thick to stop radiation. Due to the attenuation of radiation as it travels through a narrower zone, the radiation shielding performance improves with a decrease in either parameter for a given photon energy.

**Figure 8:**
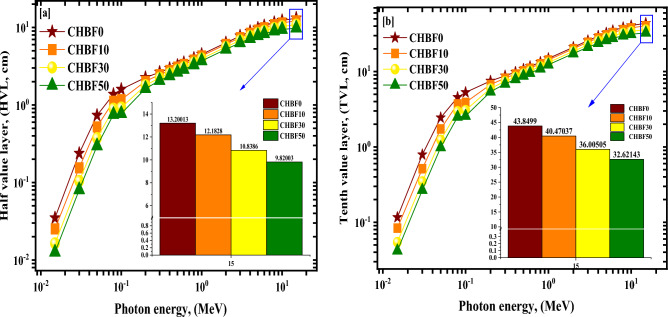
(**a–c**): (**a**) The half value layer (HVL), (**b**) the tenth value layer (TVL), and (**c**) the mean free path (MFP) for the synthetic CHBFX mortar samples versus the photon energy.

The HVL of the synthesized CHBFX mortar samples increased as the values of µ decreased because of the opposite correlation between µ and HVL_._ The HVL values grew from 0.035 to 13.200 cm^−1^ for CHBF0, from 0.025 to 12.183 cm^−1^ for CHBF10, from 0.016 to 10.839 cm^−1^ for CHBF30, and from 0.013 to 9.820 cm^−1^ for CHBF50 sample with raising the energy values from 0.015 MeV to 15 MeV as seen in Fig. 8a. The values of the TVL follow the same pattern as the HVL. The perovskite CHBF50 possesses the best radiation shielding properties, as seen by its low HVL values. These results indicated that increasing the doping of IF wt% increases the γ attenuation capabilities within the selected γ_e_ range, as shown in Fig. 8b.

Figure 8c represents the *MFP* of the examined CHBFX mortar samples as it varies with energy. The *MFP* values were found from 0.050 to 19.044 cm^−1^ for CHBF0, from 0.036 to 15.576 cm^−1^ for CHBF10, from 0.024 to 15.637 cm^−1^ for CHBF30, and from 0.018 to 14.167 cm^−1^ for CHBF50 sample. The *MFP* values reach the lowest values for the CHBF50 glass sample.

**Figure 9:**
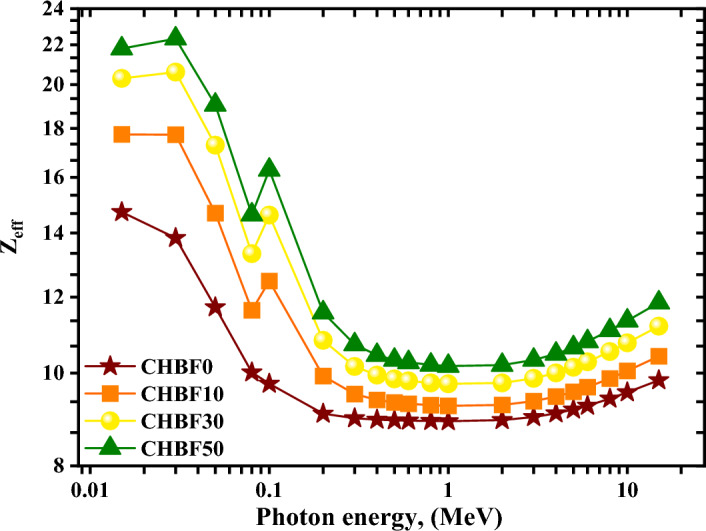
The effective atomic number (Z_eff_) for the synthetic CHBFX mortar samples versus photon energy.

Graphs of the effective atomic number (Z_eff_) vs photon energy from 0.015 to 15 MeV for the synthesis mortar samples are shown in Fig. 9. A better ability to interact with radiation, especially in the CSE and PEE zones, is indicated by larger Z_eff_ values. To protect against high-energy radiation, materials with a higher Z_eff_ value may be preferable^52–54^. The Z_eff_ values for the examined materials decline with increasing MeV. For the energy spectrum of interest, the range of Z_eff_ of the CHBFX mortar samples varied from 14.722 to 9.828 for CHBF0, from 17.744 to 10.408 for CHBF10, from 20.298 to 11.190 for CHBF30, and from 21.809 to 11.840 for CHBF50 sample. It suggests that the efficiency of the materials in radiation attenuation varies with the energy of the radiation, with a particular material perhaps being more successful at higher or lower MeV. The CHBF50 sample exhibits the highest Z_eff_ values at MeV values between 0.015: 15 MeV**.**

**Figure 10:**
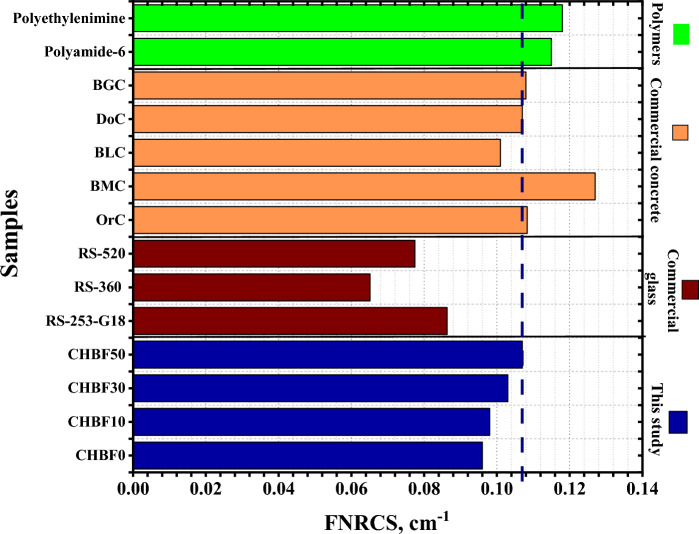
Comparison of the fast removal cross-section (FNRCS) for the CHBFX mortar samples and other commercial glass, concrete, polymer samples.

**Figure 11:**
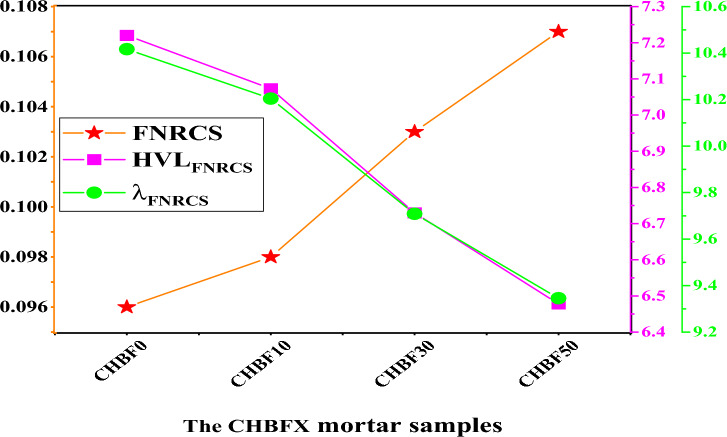
The fast neutron removal cross-section (FNRCS), the half value layer (HVL_FNRCS_), and the relaxation length (λ_FNRCS_) for the CHBFX mortar samples.

The data presented demonstrates that the fast neutrons removal cross-section (FNRCS, Σ_R_) for the four mortar samples, CHBFX, where x = 0, 10, 30, and 50 wt% were 0.096 cm^−1^, 0.098 cm^−1^, 0.103, and 0.107 cm^−1^ respectively. The highest effective removal cross-section was achieved for the CHBF50 due to the high concentration of the Oxygen light element. Also, Also, the FNRCS for the prepared mortar samples were compared with commercial glass samples, RS-253-G18, RS-360, and RS-520 as well as seven commercial concrete compounds; Ordinary (OrC), Basalt-magnetite concrete (BMC), traditional concrete mix (DoC), limonite/sand concrete (BLC), and goethite/sand/boron carbide concrete mix (BGC), and some polymers; Polyethylenimine (PEI), and Polyamide-6^48,55–58^ as seen in Fig. 10. The FNRCS value of the CHBF50 sample was found higher than the compared commercial glasses and BLC concrete sample. We can assume that the synthesized CHBFX mortar samples under investigation have better neutron shielding. Figure 11 also displays the HVL_FNRCS_ and λ_FNRCS_ for the prepared CHBFX mortar sample. Based on the simulated FNRCS values, the HVL_FNRCS_ and λ_FNRCS_ values were the lowest for the CHBF0 sample and highest for the CHBF50 mortar sample.

## Conclusion

This study inspects the gamma rays and neutron protection features of mortar composition with different percentages of iron filling. The prepared mortar samples were coded as CHBFX where x = 0, 10, 30, and 50 wt%. The results showed that the increase of iron filling concentration doping, increase the attenuation coefficient as follows:

- The $\upmu$ order is: CHBF0 < CHBF10 < CHBF30 < CHBF50
- The HVL varies inversely to the linear attenuation coefficient. Thus, CHBF50 has the lowest HVL_,_ TVL, and MFP.
- Within the investigated photon energy range, ${{\text{Z}}}_{{\text{eff}}}$ changes within the range: 14.722 to 9.828 for CHBF0, from 17.744 to 10.408 for CHBF10, from 20.298 to 11.190 for CHBF30, and from 21.809 to 11.840 for CHBF50 sample.
- The FNRCS of the CHBFX mortar samples have values of 0.096 cm^−1^, 0.098 cm^−1^, 0.103, and 0.107 cm^−1^ for the mortar samples CHBF0, CHBF10, CHBF30, and CHBF50, respectively. The values of FNRCS showed a steady increase with the increased densities of iron of the prepared mortar CHBFX samples.
- Thus, the synthetic CHBF50 mortar sample provides the best protection against gamma rays and fast neutrons.

## Data Availability

All data generated or analyzed during this study are included in this published article.
